# Supplementary material for: Content-rich biological network constructed by mining PubMed abstracts
Source: BMC Bioinformatics. 2004 Oct 8;5:147. doi: 10.1186/1471-2105-5-147 (PMC528731; doi:10.1186/1471-2105-5-147)
Supplement: Additional File 2 — The original results of the above study (non-essential files are deleted to keep the file size under the limit set by BMC bioinformatics). [file 1471-2105-5-147-S2.bz2 › chilibotAdditionalFile2/dip05/459ID10488147E10477/html/CX3CL1_CX3CR1.html]

 


 **CX3CL1** and **CX3CR1** 
  
Found 71 abstracts in PubMed, retrieved 05.  
 

 What does Google say? 
 PDF only 
| .edu only 

---

**Interactive relationship** (e.g. stimulation, inhibition, etc)

**Neutral relationship**- In contrast to CD16 monocytes, CD16 monocytes expressed high  **CX3CR1**  and CXCR4 but low CCR2 and CD62L levels and underwent efficient transendo thelial migration in response to fractalkine  [ **CX3CL1** ]  FKN.  Ref: 12810688 J Exp Med, 2003

**Non-interactive relationship** (e.g. studied together, co-existance, homology, etc.)

- Consistent with this, intravenous injection of a single dose of recombinant IL 15 into C57BL 6 mice decreased steady state  **CX3CR1**  levels 24 hours after injection in freshly isolated PBMCs, splenocytes and bone marrow cells, and treatment of mouse PBMCs with IL 15 in vitro inhibited  **CX3CL1**  induced chemotaxis.  Ref: 12881312 BloodBlood, 2003
- Interactions between fractalkine  [ **CX3CL1** ]  and  **CX3CR1**  can mediate not only chemotaxis but also cell adhesion in the absence of substrates for other adhesion molecules.  Ref: 12806431 Drug News Perspect, 2001
- Both fractalkine  [ **CX3CL1** ]  and  **CX3CR1**  were expressed constitutively in neurons, microglia, and astrocytes.  Ref: 12850572 Brain Res, 2003
- Fractalkine  [ **CX3CL1** ]  receptor  **CX3CR1**  is expressed on natural killer NK cells, monocytes and some portions of CD8 T cells.  Ref: 12806431 Drug News Perspect, 2001
- OBJECTIVESWe have recently reported that the polymorphism of the fractalkine  [ **CX3CL1** ]  receptor,  **CX3CR1** , provides a new marker for prognosis in HIV disease.  Ref: 12799549 AIDS, 2003
- In the present study, we examined the production of fractalkine  [ **CX3CL1** ]  and the expression of its receptor,  **CX3CR1**  in murine glial and neuronal cell in vitro, and investigated its neuroprotective functions.  Ref: 12850572 Brain Res, 2003
